# Evaluation of the Quebec Healthy Enterprise Standard: Effect on Adverse Psychosocial Work Factors and Psychological Distress

**DOI:** 10.3390/ijerph15030426

**Published:** 2018-02-28

**Authors:** Marie-Claude Letellier, Caroline S. Duchaine, Karine Aubé, Denis Talbot, Marie-Michèle Mantha-Bélisle, Hélène Sultan-Taïeb, France St-Hilaire, Caroline Biron, Michel Vézina, Chantal Brisson

**Affiliations:** 1 Faculty of Medicine, Laval University, Quebec City, QC G1V 0A6, Canada; marie-claude.letellier.1@ulaval.ca (M.-C.L.); caroline.duchaine@crchudequebec.ulaval.ca (C.S.D.); denis.talbot@fmed.ulaval.ca (D.T.); 2 Institut National de santé Publique du Québec, Quebec City, QC G1V 5B3, Canada; marie-michele.mantha-belisle@inspq.qc.ca (M.-M.M.-B.); michel.vezina@fmed.ulaval.ca (M.V.); 3 Population Health and Optimal Health Practices Unit, CHU de Quebec Research Center, Quebec City, QC G1S 4L8, Canada; 4 School of Management Sciences, University of Quebec in Montréal, Montreal, QC H2X 3X2, Canada; sultan_taieb.helene@uqam.ca; 5 Management School, University of Sherbrooke, Sherbrooke, QC J1K 2R1, Canada; france.st-hilaire@usherbrooke.ca; 6 Faculty of Administration Sciences, Laval University, Quebec City, QC G1V 0A6, Canada; caroline@prevacsult.com

**Keywords:** workplace intervention, psychological distress, mental health, occupational health standard, standard certification, psychosocial work factors, sick leave, management practices, work stress

## Abstract

Adverse psychosocial work factors are recognized as a significant source of psychological distress, resulting in a considerable socioeconomic burden. The impact of occupational health standards that aim to reduce these adverse work factors, such as the Quebec Healthy Enterprise Standard (QHES), is of great interest for public health. The aim of this study was to evaluate, for the first time, the effect of QHES interventions targeting adverse psychosocial work factors on the prevalence of these factors and of psychological distress among ten Quebec organizations. These outcomes were assessed by questionnaire using validated instruments before (T1, n = 2849) and 2–3 years following (T2, n = 2560) QHES implementation. Beneficial effects of interventions were observed for two adverse psychosocial work factors: low rewards (ratio of prevalence ratios (PRs) = 0.77, 95% CI = 0.66–0.91) and low social support at work (ratio of PRs = 0.89, 95% CI = 0.77–1.03). Moreover, beneficial effects of interventions were also observed on the prevalence of high psychological distress (ratio of PRs = 0.86, 95% CI = 0.75–0.998). Psychosocial interventions implemented in the context of this standard improved the psychosocial work environment and had beneficial effects on workers’ mental health.

## 1. Introduction

Mental health problems are a major cause of disability worldwide [1]. An estimated 25% of the European population are affected by a mental health problem each year [2] and more than 6.7 million people in Canada were living with a mental health problem in 2011 [3]. In industrialized countries, these problems are the first or second leading cause of sick leave from work [4,5,6]. Moreover, they place a considerable burden on society and present an important source of loss of productivity for employers [5,7,8]. Psychological distress is widely used as an indicator of mental health in epidemiological studies [9]. It is generally defined as a state of emotional suffering characterized by depressive and anxious symptoms [9]. Psychological distress is associated with diagnosed mental health disorders in the general population [10,11,12] and is prospectively associated with sickness absence for mental health problems among workers [13,14]. Given that up to 25% of Canadian and American workers from various industries experience high psychological distress [15,16], workplace interventions aiming to prevent and reduce it are of important interest for public health. 

Adverse psychosocial work factors are recognized as a significant source of psychological distress and an important contributing factor in the development of mental health problems [17,18,19,20]. Primary preventive interventions at the workplace aiming to reduce these factors may thus help improve the mental health of workers [21]. In this field, the predominant adverse psychosocial work factors identified in the literature stem from two internationally recognized models: the demand-control-support (DCS) [22,23] and effort-reward imbalance (ERI) [24] models. These models refer to the organizational and interpersonal factors of a workplace that may negatively impact workers’ health such as high psychological demands, low decision latitude, low social support at work and low rewards [22,23,24]. Available evidence suggests that it is possible to reduce workers’ exposure to these adverse psychosocial work factors through workplace organizational interventions and that these interventions may lead to improvements in mental health indicators [21,25,26,27,28,29,30,31,32,33]. This highlights the importance and potential of this type of strategy for the primary prevention of mental health problems.

For these reasons, adverse psychosocial work factors have harbored the attention of policy-makers, industry experts and key mental health organizations [34,35,36,37,38,39,40]. At a national level, Canada has developed two voluntary standards that emphasize the reduction of adverse psychosocial work factors to improve the health of the working population [41,42]. Among these, only the Quebec standard “Prevention, Promotion and Organizational Practices Contributing to Health in the Workplace” (BNQ 9700-800/2008), commonly called the Quebec Healthy Enterprise Standard (QHES), leads to a certification [41]. Organizations implementing this voluntary occupational health standard must follow a comprehensive implementation process elaborated by the provincial standard association and respect certain pre-established conditions in order to be awarded certification. In contrast with other standards, this certification process provides an objective measure of implementation through an external audit [41,43]. Interventions implemented as part of the QHES target four areas of activity to improve employees’ physical and mental health: (i) Lifestyle Habits; (ii) Work-life Balance; (iii) Physical Environment; and (iv) Management Practices. Interventions implemented within this latter area include activities that target adverse psychosocial work factors. As such, the implementation of the QHES may help reduce these adverse occupational exposures and thus improve the mental health of workers.

The effect of this innovative occupational health standard on workers’ mental health has not yet been evaluated. In addition, and to our knowledge, no previous evaluation of the mental health effects of a standard of this type has been conducted to date. Given the social and economic burden associated with mental health problems and the role of adverse psychosocial work factors in their development, it is pertinent to assess the impact of the QHES in reducing these adverse work factors and improving the mental health of workers. The aim of this study was to evaluate the effects of interventions implemented in the Management Practices area of the QHES on the prevalence of adverse psychosocial work factors and of psychological distress among workers of ten Quebec organizations.

## 2. Materials and Methods

### 2.1. QHES Implementation and Certification Process

The implementation of the QHES is a participatory process that requires management engagement, the creation of a health and well-being committee composed of both managers and employees, a needs assessment, and an on-going record of implemented intervention activities [43]. For the needs assessment, data must be collected regarding employees’ needs and risk exposures related to the four QHES areas of activity. These must be compiled confidentially and updated at least once every two to three years. There is no standardized method to collect this data; each organization can rely on its own expertise or ask an external expert to collect data. The compiled results of this data are used by the health and well-being committee as a needs assessment to guide intervention activities.

Quebec’s standard association, the Bureau de Normalisation du Québec (BNQ), is responsible for the QHES certification process and performs external audits to verify QHES implementation [43]. The BNQ first reviews the internal documents and records that the organization has collected in the context of implementing this standard and conducts interviews with the organization’s executives, health and well-being committee members, program managers and regular employees. Second, the BNQ compiles and delivers a report to the organization that includes the organization’s strengths, opportunities for improvement as well as a list (if any) of changes that need to be made to obtain the certification. Once all conditions are met, the organization receives its certification for a two to three-year period and an audit must be undertaken each year by the BNQ to verify QHES implementation. After this period, and to obtain a renewal, a re-certification process must be undertaken by the organization with the BNQ [43].

### 2.2. Interventions in the Management Practices Area of the QHES

As one of the four areas of activity of the QHES, the Management Practices area is defined by the standard as all the managerial and organizational practices and methods of work modality [41]. As suggested by the QHES [41], this can include tools and support available for workers to perform their work tasks, recognition programs, facilitating communication between superiors and workers, employee participation in decision-making and providing workers with career development and training tools [41]. Together with management, each organization’s health and well-being committee proposes and implements interventions based on the compiled results of the aforementioned needs assessment. As such, each organization implements interventions tailored to their needs and context. 

### 2.3. Study Design and Population

This is a before-after design with a reference group. Organizations that were involved in the certification process at the study’s conception and who requested that the Institut National de Santé Publique du Québec (INSPQ) collect data for their needs assessment were eligible for the present study. All eligible organizations (N = 10) were invited to participate and all ten agreed to participate in the study. These organizations were among the first to implement the standard and hailed from the public (7/10) and private (3/10) sectors, covering a wide spectrum of economic activities in the province of Quebec; namely in public administration (8/10), but also in the manufacturing (1/10) and banking (1/10) sectors. The size of the organizations varied from 103 to 1467 workers. All participating organizations implemented the QHES between May 2011 and December 2013 (T1) and completed a follow-up between May 2014 and November 2015 (T2). The period between T1 and T2 ranged from two to three years during which QHES interventions were implemented. All workers (both active and on leave) were solicited by their employer to participate. The final sample included 2849 workers at T1 (67–90% participation) and 2560 workers at T2 (63–88% participation). Participants occupied roles as executives, professionals, technicians, office staff and manual staff.

### 2.4. Data Collection and Measures

Employees of the ten participating organizations completed a questionnaire developed by the INSPQ for the purpose of evaluating the QHES. This 30-min self-report questionnaire was administered at the workplace during work hours and included a variety of items to assess exposure to workplace risk factors and several health outcomes. A second section was added to the T2 questionnaire by the research group to assess participants’ exposure to QHES interventions, as described in the next section. The INSPQ was responsible for data collection.

#### 2.4.1. Intervention Exposure in the Management Practices Area of the QHES

In the T2 questionnaire, we evaluated participants’ perceived exposure to interventions in the Management Practices area of the QHES. Participants were asked to rate if they had observed changes in their workplace since the implementation of the QHES regarding (1) workload, (2) autonomy, (3) support from colleagues and superiors and (4) recognition. These items were adapted from a questionnaire previously used by our research group [44] and reflect the four main psychosocial work factors identified by the DCS and ERI models. Participants were considered exposed to interventions if they responded, on any of the five items, that the changes they perceived in their workplace “improved”, “did not change”, or “deteriorated” their work situation. Participants were considered not exposed to interventions if they answered either “no change implemented”, or “I do not know” to all five items. If all five items were rated as “I do not know”, it was treated as a non-response. The internal consistency (Cronbach’s alpha) of these five items was 0.89. This instrument was used for seven organizations which employed, collectively, 86% of the study population. For the three remaining organizations (14%), one general item was used to assess participants’ perceived exposure to interventions in this area, as described in Appendix A Table A1. Intervention exposure was treated dichotomously and continuously in the analyses (see Section 2.5).

#### 2.4.2. Adverse Psychosocial Work Factors

Psychological demands were assessed with five items from the short French version of the Job Content Questionnaire (JCQ) [45] and one item from the full version of the JCQ, “My tasks are often interrupted before they can be completed” [46]. Decision latitude was evaluated with five items adapted from the JCQ [46]. Social support at work was evaluated with six items from the French version of the JCQ [45] in addition to one item from the Copenhagen Psychosocial Questionnaire (COPSOQ), “At my work, I have the impression to be part of a team” [47]. The psychometric properties of these instruments have been demonstrated [48,49]. Reward was measured using six items from the validated ERI Questionnaire [50,51] with the addition of two other validated items from the COPSOQ, “At work, my efforts are adequately appreciated” and “At work, I am treated fairly” [47]. In the present study the internal consistency of each scale, as measured by the Cronbach’s α coefficient, was 0.74 for psychological demands, 0.67 for decision latitude, 0.81 for social support at work and 0.75 for rewards. Each item was rated on a four-point Likert scale (0–3). The median observed in a representative sample of the Quebec working population [52] was used to dichotomize exposure to high psychological demands (score > 9), low decision latitude (score < 24), low social support at work (score < 51) and low rewards (score < 15). Exposure to job strain was defined as a combination of high psychological demands and low decision latitude [45,46]. The ERI ratio was calculated by dividing the score of psychological demands (proxy for effort) by the score of rewards. Participants with an ERI ratio higher than one were considered exposed to ERI [50,51].

#### 2.4.3. Psychological Distress

Psychological distress was evaluated with the K6, a 6-item validated scale [12,53] developed by Kessler [10,11]. This instrument is widely used in national surveys and epidemiological studies as an indicator of mental health [9,52,54]. Participants rated on a five-point Likert-type scale (0–4) ranging from all the time to none of the time how often, in the past 30 days, they had felt nervous, hopeless, restless, so depressed that nothing could cheer them up, that everything was an effort and worthless. A sum of scores was computed and, as recommended, the score was dichotomized; a score of seven or higher represented high psychological distress [10,11].

#### 2.4.4. Control Variables

Several sociodemographic variables were used as covariates: sex, age (<45, 45–54, ≥55), education (high school degree or less, college degree, university degree), physical activity (frequency per week <3, ≥3), smoking status (non-smoker, smoker) and fruit and vegetable intake (servings/day <5, ≥5).

### 2.5. Statistical Analyses

First, to verify the theoretical assumption that adverse psychosocial work factors are associated with psychological distress in the present sample, we conducted a cross-sectional analysis among the 2849 participants at T1. Prevalence and prevalence ratios (PRs) and their 95% confidence intervals (CI) of high psychological distress according to exposure to adverse psychosocial work factors were estimated using generalized estimating equations with log-link and binomial distribution.

Due to privacy issues, participants’ pairing information was not available. Consequently, it was not possible to compare how the individual outcomes changed from T1 to T2 according to participants’ level of exposure to interventions in the Management Practices area of the QHES. Instead, this comparison was performed at the organizational level; that is the data were aggregated at the organizational level at each time-point. First, organizations were categorized as either more exposed or less exposed to these interventions as follows: (1) the proportion of participants who were considered exposed to interventions in the Management Practices area at T2 were calculated for each organization; and (2) the five organizations with the highest proportions of participants exposed (78%, 79%, 80%, 86%, 88%) were classified as more exposed to interventions in this area and the five organizations with the smallest proportions of participants exposed (60%, 71%, 72%, 76%, 76%) were classified as less exposed. Second, the proportion of participants exposed to interventions in the Management Practices area was also treated as a continuous variable.

Two sets of outcome variables were examined: adverse psychosocial work factors and psychological distress. These were also aggregated at the organizational level. At each time-point, the outcome was summarized as the number of individuals reporting a given outcome variable in an organization divided by the total number of participants in that organization. Covariates were treated in the same fashion.

First, PRs and their 95% CI comparing the change from T1 to T2 within exposure group were estimated using repeated measure log-binomial regressions fitted on the organizational level data. The outcome data was entered in an “events/trials” fashion, thus accounting for the number of participants in each organization at each time-point. The correlation between repeated measures was accounted for by utilizing a compound symmetry matrix. The ratios of PRs (PR of more exposed organizations/PR of less exposed organizations) along with their 95% CI and the test for group by time interaction were used to compare the change in the prevalence of the outcome from T1 to T2 between organizations more and less exposed to interventions. Second, an analysis where the proportion of participants exposed to interventions was entered as a continuous variable in the log-binomial model instead of being categorized was performed. The test for the interaction term between the proportion of participants exposed and time was used to assess if PRs varied according to the proportion of participants exposed. The correlation between repeated measures was accounted for using a robust variance estimator in this analysis [55]. These analyses provide the net effect of the interventions and also control for baseline and time-invariant potentially confounding characteristics of the organizations.

Analyses were performed to assess potential confounding. The reduced sample size resulting from aggregating data at the organizational level did not allow us to include all variables in the same model, but we tested all possible combinations. First, for all the outcomes, analyses were adjusted for sociodemographic factors (age, sex, and education). Second, the analyses with psychological distress were further adjusted for lifestyle habits (smoking status, physical activity and fruit and vegetable intake).

Sensitivity analyses were also performed to test the robustness of the results. First, analyses for all outcomes were performed among the seven organizations that used the more complete measure of intervention exposure. This was done in order to verify if the difference in the questionnaire used to measure the intervention exposure could have an impact on the effect estimates. In addition, the three organizations most exposed to interventions were compared to the three organizations that were the least exposed in order to determine if the results were influenced by a change in the categorization of intervention exposure.

SAS^®^ 9.4 software (SAS Institute Inc., Cary, USA) [56] was used for all analyses. Participants who did not adequately complete the questionnaire were only excluded from the specific analyses where their data were missing. Missing data were present in less than 6% of participants for any given variable.

### 2.6. Ethical Considerations

This project was approved by the Research Ethics Committee of the University of Québec in Montreal, the Comité Institutionnel d’Éthique de la Recherche avec des Êtres Humains (S-7034324) and the Research Ethics Committee of the CHU de Québec Research Center, the Comité d’Éthique de la Recherche du CHU de Québec-Université Laval (108812). Informed consent was obtained from all participants. Data was made available to the present research team after consent was obtained from all participating organizations. Denominalized databases are stored under password protection on a secure network.

## 3. Results

A description of the study population before QHES implementation is presented in Table 1. There were an equal proportion of men and women in our sample and the majority of participants were between 25 and 54 years old. The highest level of education completed for the majority of participants was a college or university degree. Participants from organizations more exposed to interventions in the Management Practices area of the QHES had a lower educational attainment, a lower frequency of physical activity per week, a higher prevalence of smoking and a lower intake of fruits and vegetables than participants in less exposed organizations.

The prevalence and PRs of high psychological distress according to exposure to adverse psychosocial work factors before QHES implementation are presented in Table 2. The prevalence of high psychological distress was consistently associated with exposure to adverse psychosocial work factors, with adjusted PRs ranging from 1.34 to 2.02 (*p* < 0.0001). All associations reached statistical significance, even after adjustment for sociodemographic factors and both sociodemographic factors and lifestyle habits.

The prevalence and PRs of adverse psychosocial work factors according to organizations’ intervention exposure as a dichotomous variable are presented in Table 3. After QHES implementation, organizations more exposed to interventions had decreases in the prevalence of two adverse psychosocial work factors: low social support at work (PR = 0.87, 95% CI = 0.77–0.98) and low rewards (PR = 0.86, 95% CI = 0.74–0.99). Among organizations less exposed to interventions, an inverse tendency was observed. Indeed, these organizations had increases in the prevalence of three adverse psychosocial factors: high psychological demands (PR = 1.11, 95% CI = 1.02–1.22), low rewards (PR = 1.11, 95% CI = 1.02–1.21) and ERI (PR = 1.17, 95% CI = 1.06–1.30). The net effect of interventions, as measured by the ratios of PRs, indicated decreases due to interventions for two adverse factors: low rewards (ratio of PRs = 0.77, 95% CI = 0.66–0.91, *p* = 0.007) and ERI (ratio of PRs = 0.80, 95% CI = 0.64–0.99, *p* = 0.048). The net effect of interventions also suggested a decrease in the prevalence of low social support at work, although it did not reach statistical significance (ratio of PRs = 0.89, 95% CI = 0.77–1.03, *p* = 0.099). Similar tendencies were observed in the analyses adjusted for age, sex, and education (see Table S1), but the CIs were larger, as expected in this kind of analysis.

The prevalence and PRs of high psychological distress according to organizations’ intervention exposure as a dichotomous variable are presented in Table 4. After QHES implementation, organizations more exposed to interventions had a decrease in the prevalence of high psychological distress (PR = 0.83, 95% CI = 0.73–0.93), whereas little change was observed in less exposed organizations (PR = 0.96, 95% CI = 0.88–1.03). The net effect of interventions showed a 14% decrease in the prevalence of high psychological distress (ratio of PRs = 0.86, 95% CI = 0.75–0.998, *p* = 0.048). A similar net effect was observed in the analyses adjusted for age, sex, and education (see Table S2), but the CIs were larger, as expected in this kind of analysis.

**Figure 1:**
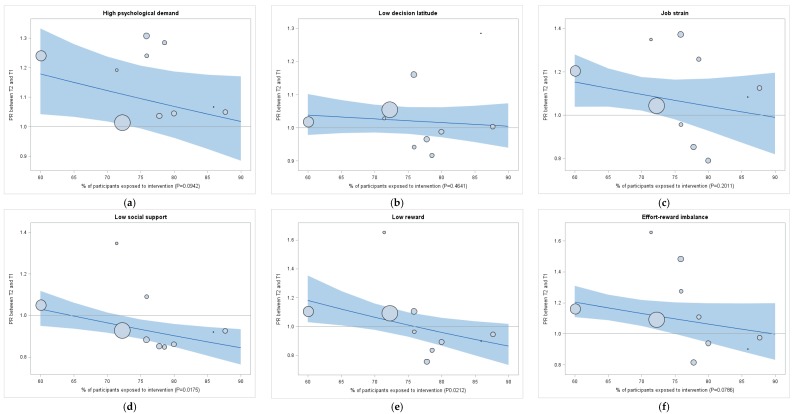
Proportion (%) of participants exposed to interventions in the Management Practices area of the QHES ^1^ as a continuous variable: Prevalence ratios (PR) of adverse psychosocial work factors before (T1) and after (T2) QHES ^1^ implementation for each organization. ^1^ QHES= Quebec Healthy Enterprise Standard; CI = Confidence interval; ERI = Effort-reward imbalance; Prevalence ratios (PR) of adverse psychosocial work factors before (T1) and after (T2) QHES implementation. PRs were estimated with a repeated measure log-binomial regression where the proportion (%) of participants exposed to interventions in the Management Practices area of the QHES was considered as a continuous variable, non-adjusted. Grey bands represent 95% confidence intervals. The sizes of the bubbles are proportional to the number of participants in each organization. The horizontal line separates the results between organizations where the prevalence was higher at T2 than at T1 (above the line) and organizations where the prevalence was lower at T2 than at T1 (below the line). *p*-values are for exposition*time interaction test. (**a**) PR of high psychological demand between T2 and T1; (**b**) PR of low decision latitude between T2 and T1; (**c**) PR of job strain between T2 and T1; (**d**) PR of low social support between T2 and T1; (**e**) PR of low reward between T2 and T1; (**f**) PR of ERI between T2 and T1.

**Figure 2:**
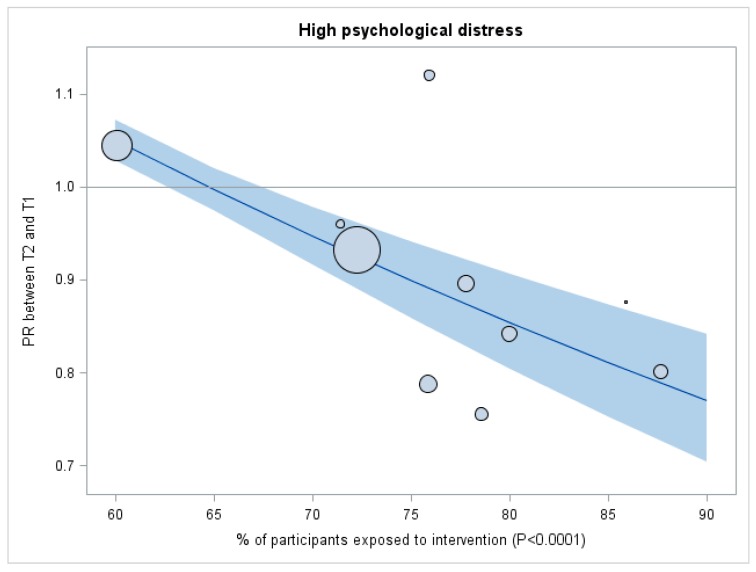
Proportion (%) of participants exposed to interventions in the Management Practices area of the QHES ^1^ as a continuous variable: Prevalence ratios (PR) of high psychological distress before (T1) and after (T2) QHES ^1^ implementation for each organization. ^1^ QHES = Quebec Healthy Enterprise Standard; CI = Confidence interval; Prevalence ratios (PR) of high psychological distress before (T1) and after (T2) QHES implementation. PRs were estimated with a repeated measure log-binomial regression where the proportion (%) of participants exposed to interventions in the Management Practices area of the QHES was considered as a continuous variable, non-adjusted. Grey bands represent 95% confidence intervals. The size of the bubbles are proportional to the number of participants in each organization. The horizontal line separates the results between organizations where the prevalence was higher at T2 than at T1 (above the line) and organizations where the prevalence was lower at T2 than at T1 (below the line). *p*-values are for exposition*time interaction test.

The results of the analyses where intervention exposure is a continuous variable are presented in Figure 1 for adverse psychosocial work factors and in Figure 2 for psychological distress. The PR between T1 and T2 for low social support and for low reward decreased when the proportion of participants exposed to the interventions increased (*p*-value for exposition*time interaction test = 0.0175 and 0.0212 respectively). As shown in Figure 1, organizations less exposed to interventions showed an increase in these adverse psychosocial work factors between T1 and T2 (above the line of PR = 1.00) compared to organizations more exposed to interventions; where the prevalence of adverse psychosocial work factors tended to decrease between T1 and T2 (below the line of PR = 1.00). As shown in Figure 2, similar results were observed for psychological distress (*p*-value for exposition*time interaction test <0.0001). Results adjusted for age, sex and education are presented in Figure S1 for adverse psychosocial work factors and in Figure S2 for psychological distress. The results were similar, but statistical significance was only reached for psychological distress (*p*-value for exposition*time interaction test = 0.0081).

The results of the sensitivity analyses are presented in the Supplementary files (Tables S3–S6). Results were similar for all adverse psychosocial work factors and for psychological distress among the seven organizations that used the more complete measure of intervention exposure (Tables S3 and S4). Analyses among the three organizations most exposed to interventions compared to the three organizations least exposed to interventions also showed results similar to the main analyses (Tables S5 and S6). Therefore, the general trend of these sensitivity analyses corroborates our aforementioned findings in the main analyses, which used a dichotomous and continuous variable of intervention exposure (Table 3 and Table 4, Figure 1 and Figure 2).

## 4. Discussion

This study used a before-after design with a reference group to evaluate the effects of interventions implemented in the Management Practices area of the Quebec Healthy Enterprise Standard (QHES); an area that targets the psychosocial work environment. Following implementation of this standard, beneficial effects were observed. There was a decrease in the prevalence of two adverse psychosocial work factors among organizations that were more exposed to interventions in the Management Practices area, namely low social support at work and low rewards. There was also a decrease in the prevalence of high psychological distress among these organizations. 

These results suggest that organizational interventions implemented in the context of the Management Practices area of the QHES improved the psychosocial work environment and had beneficial effects on workers’ mental health. These results parallel the findings of other intervention studies showing that psychosocial workplace interventions can reduce workers’ exposure to adverse psychosocial work factors and improve their mental health [21,25,26,27,28,29,30,31,32,33,57,58,59,60]. 

To our knowledge, this study is the first to evaluate the mental health effects of an occupational health standard with a certification process and an external audit. The results suggest that the participating organizations together with their health and well-being committees recognized the pertinence of intervening in the Management Practices area of the QHES. This was not only demonstrated by our main results regarding the beneficial effects of to these interventions in reducing the prevalence of adverse psychosocial work factors and high psychological distress, but also by the proportion of participants that considered themselves exposed to interventions in this area of activity (between 60–88%). These results are especially encouraging considering that the Management Practices area is one of four potential areas of intervention proposed by the QHES, aside from Lifestyle Habits, Physical Environment, and Work-life Balance.

The findings presented here show that social support and rewards improved in more exposed organizations but psychological demands and decision latitude remained unchanged. This suggests that organizations were perhaps more willing to improve social support and rewards through a number of social activities within the organization than to change psychological demands and decision latitude; these latter psychosocial work factors inherently implicate aspects of productivity, work organization as well as employers’ management rights that may not be as easily targeted for change than aspects related to the social work environment.

It is of note that the QHES is currently undergoing a revision process and that, among employers, improving the mental health of workers is an important priority given the high frequency of mental health problems and their associated costs due to loss of productivity, presenteeism, long absences from work and important compensation costs. The current results have been presented in several knowledge transfer activities between our research team and members of the QHES revision committee. Our findings have demonstrated that it is possible to reduce adverse psychosocial work factors and psychological distress, thereby improving the mental health of workers. In the context of revising the QHES, our findings respond to a priority of employers and highlight the benefits of QHES implementation. Together with the revision of the standard and the increased awareness among employers regarding the benefits of intervening in the Management Practices area, this could lead to QHES intervention activities expanding to target aspects related to psychological demands and decision latitude. Our previous intervention studies have shown that interventions related to psychological demands and decision latitude are feasible and have been implemented in a number of work settings [21].

Very few studies [61] have attempted to evaluate the implementation process in the context of national standards. Organizational interventions have often been studied using designs that do not account for natural variations in exposure to intervention activities [62]. This limits the possibilities to explain why an intervention fails or succeeds [63]. Considering the implementation processes and the natural variations in exposure to interventions increases the validity of the study since it helps the researcher to rule out competing hypotheses by contrasting groups of participants who were naturally more and less exposed to the intervention [57,62,64]. This is especially relevant given the complex and uncontrollable nature of organization-wide interventions. In the current study, a before and after study design with a reference group was used. To define the reference group, post-hoc measures of intervention exposure were used so that the evaluation can take into account whether each participant was exposed or not exposed to interventions. This allowed us to capture natural variations in exposure levels to interventions. This type of alternative research design is particularly useful when a classic control group and randomization are not an option [65] and the conditions for a true experiment cannot be met [64].

This study has several strengths. First, the number of participants in each organization was relatively high and the sample was composed of a similar proportion of men and women. Second, a diversity of occupations and types of organizations were represented in our sample. Third, QHES interventions in the Management Practices area were based on validated conceptual models reflecting the four main psychosocial work factors identified by the DCS and ERI models. Fourth, validated instruments were used to evaluate adverse psychosocial work factors and psychological distress. Fifth, this is the first evaluation, to our knowledge, of a voluntary occupational health standard that includes a certification process. This certification process provides an objective measure of implementation through an external audit. 

This study has also limitations. First, the absence of a completely unexposed group did not allow us to completely isolate the interventions’ effect. Indeed, the reference group was also exposed to some extent (the difference in the proportion of participants exposed to interventions was small between some organizations belonging to different exposure groups). This might have limited our ability to observe between-group outcome differences and lead to an underestimation of the true effects. However, analyses with intervention exposure as a continuous variable were also conducted and the results showed a similar tendency. Moreover, sensitivity analyses conducted by using the three organizations most and least exposed to interventions also showed similar results (Tables S5 and S6), although these did not reach statistical significance (likely due to diminished statistical power resulting from analyses with six organizations instead of ten). Second, a different measure of intervention exposure was used in the T2 questionnaire for three organizations. Given that a different exposure measure could impact the estimates, we conducted sensitivity analyses with the seven organizations that used the more complete exposure measure; the estimates obtained were similar to those of the main analyses (Tables S3 and S4) therefore showing that differences in questionnaire formulation did not influence the results. Third, this study relied on self-report data on exposure to interventions. This could lead to an underestimation or an overestimation of the true effect given that participants could erroneously consider an organizational change as part of the QHES when, in reality, it was not, or vice versa. Fourth, given that the outcomes assessed were all self-reported, it is possible that participants’ awareness of being part of an intervention study could have influenced their responses on the questionnaire. However, both groups were subject to this potential factor as they were both part of the QHES implementation process and intervention study. Therefore, this factor is unlikely to explain the beneficial effects observed in the intervention group. Fifth, due to our inability to pair participants’ responses between T1 and T2, we were unable to perform the analyses at the individual level. Instead, data were aggregated at the organizational level, which resulted in diminished statistical power. Although this could be a limitation, analyses were performed treating intervention exposure as both a dichotomized and a continuous variable using a robust estimator and all results showed the same tendency. Aggregation of the data also limited our ability to perform adjustments for potential confounders at the individual level. However, our repeated measures models control for baseline and time-invariant characteristics of the organizations by design. Moreover, the estimates obtained in our analyses where adjustments were performed for potential confounders at the organizational level were very similar to those of the main analyses (Tables S1 and S2), therefore supporting our confidence in the results observed in the main analyses. Finally, the intervention effects could not be completely attributable to interventions in the Management Practices area. Due to the rigorous QHES certification process, organizations had to implement QHES activities in at least two areas and were obligated to implement interventions in the Lifestyle Habits area. It is thus difficult to isolate interventions according to the Management Practices area only. However, among the five organizations that were considered more exposed to Management Practices, two were also more exposed to interventions in the Physical Environment area simultaneously (data not shown) and none implemented evidence-based interventions in the Work-life Balance area. Thus, the contribution of the other QHES areas to the observed effect, if present, is likely not major.

## 5. Conclusions

This study evaluated, for the first time, the effect of interventions implemented in the Management Practices area of the QHES on the prevalence of adverse psychosocial work factors and on the prevalence of psychological distress among ten organizations. Our results demonstrate that organizational psychosocial interventions implemented in the context of this voluntary Canadian standard improved the psychosocial work environment and had beneficial effects on workers’ mental health. Given the burden associated with mental health problems and the role of certain occupational risk factors in their development, primary prevention efforts to improve workers’ mental health are relevant for both occupational and public health. Our findings provide support for the effectiveness of voluntary occupational health standards as pertinent strategies for the primary prevention of mental health problems among the working population.

## Figures and Tables

**Table 1:** Sociodemographic characteristics and lifestyle habits of the study population before QHES ^1^ implementation (T1), for all organizations combined and by organizations’ exposure to interventions in the Management Practices area of the QHES ^1^.

Sociodemographic Characteristics and Lifestyle Habits	All Organizations Combined (n = 2849)		More Exposed Organizations (n = 776)		Less Exposed Organizations (n = 2073)	
	n	%	n	%	n	%
**Gender**						
Women	1402	49.2	421	54.3	981	47.3
Men	1447	50.8	355	45.8	1092	52.7
**Age (years)**						
<25	74	2.6	13	1.7	61	2.9
25–44	1336	46.9	221	28.5	1115	53.8
45–54	947	33.3	310	40.0	637	30.7
≥55	491	17.2	231	29.8	260	12.5
**Education (highest level completed)**						
Less than high school	37	1.3	9	1.2	28	1.4
High school degree	751	26.4	375	48.5	376	18.2
College degree ^2^	1024	36.0	251	32.5	773	37.3
University degree	1033	36.3	138	17.9	895	43.2
**Frequency of physical activity per week**						
<1	425	15.0	173	22.5	252	12.2
1–2	596	21.0	182	23.6	414	20.0
3–4	1118	39.4	252	32.7	866	41.8
≥5	701	24.7	163	21.2	538	26.0
**Smoking status**						
Non-smoker	1608	56.6	341	44.3	1267	61.2
Ex-smoker	813	28.6	274	35.6	539	26.0
Occasional smoker	136	4.8	37	4.8	99	4.8
Regular smoker	283	10.0	117	15.2	166	8.0
**Fruit and vegetable intake (servings/day)**						
≤2	293	10.3	119	15.5	174	8.4
3–4	1643	57.9	467	61.0	1176	56.8
≥5	902	31.8	180	23.5	722	34.9

^1^ QHES = Quebec Healthy Enterprise Standard; ^2^ In the province of Quebec, college refers to two years of pre-university education or three years of vocational/technical training completed in addition to the five years of high school; Less than 0.01 missing values.

**Table 2:** Prevalence (%) and prevalence ratios (PR) of high psychological distress according to exposure to adverse psychosocial work factors among the 2849 participants before (T1) QHES ^1^ implementation.

Adverse Psychosocial Work Factors	Prevalence of High Psychological Distress		Crude		Model 1 ^2^		Model 2 ^3^	
	Exposed %	Unexposed %	PR (95% CI ^1^)	*p*-Value	PR (95% CI ^1^)	*p*-Value	PR (95% CI ^1^)	*p*-Value
High psychological demands	38.1	24.5	1.55 (1.42–1.70) *	<0.0001	1.58 (1.46–1.72) *	<0.0001	1.57 (1.45–1.70) *	<0.0001
Low decision latitude	32.6	23.3	1.40 (1.23–1.59) *	<0.0001	1.36 (1.20–1.55) *	<0.0001	1.34 (1.18–1.53) *	<0.0001
Job strain	41.6	25.4	1.64 (1.48–1.82) *	<0.0001	1.62 (1.45–1.81) *	<0.0001	1.59 (1.40–1.79) *	<0.0001
Low social support at work	36.3	21.3	1.70 (1.48–1.96) *	<0.0001	1.71 (1.51–1.94) *	<0.0001	1.67 (1.48–1.89) *	<0.0001
Low rewards	36.1	19.6	1.84 (1.57–2.17) *	<0.0001	1.82 (1.55–2.14) *	<0.0001	1.76 (1.52–2.05) *	<0.0001
ERI ^1^	46.3	22.7	2.04 (1.75–2.39) *	<0.0001	2.08 (1.76–2.45) *	<0.0001	2.02 (1.72–2.37) *	<0.0001

* Denotes statistical significance, *p* < 0.05; ^1^ QHES = Quebec Healthy Enterprise Standard; CI = Confidence interval; ERI = Effort-reward imbalance; ^2^ Adjusted for age (<45, 45–54, ≥55), sex, education (high school degree or less, college degree, university degree). ^3^ Adjusted for age (<45, 45–54, ≥55), sex, education (high school degree or less, college degree, university degree), smoking status (non-smoker, ex-smoker, occasional smoker and regular smoker), physical activity (frequency per week <1, 1–2, 3–4, ≥5) and fruit and vegetable intake (servings/day ≤2, 3–4, ≥5).

**Table 3:** Prevalence (%) and prevalence ratios (PR) of adverse psychosocial work factors according to organizations’ exposure to interventions in the Management Practices area of the QHES ^1^, before (T1) and after (T2) QHES ^1^ implementation.

Adverse Psychosocial Work Factors	More Exposed Organizations (n = 5)			Less Exposed Organizations (n = 5)			Net Effect of Interventions	
	T1 %	T2 %	PR (95% CI ^1^)	T1 %	T2 %	PR (95% CI ^1^)	Ratio of PRs ^2^ (95% CI ^1^)	*p*-Value ^3^
High psychological demands	33.1	36.0	1.09 (0.92–1.29)	38.1	42.4	1.11 (1.02–1.22) *	0.97 (0.81–1.18)	0.790
Low decision latitude	68.4	67.3	0.99 (0.92–1.05)	53.1	55.4	1.04 (0.99–1.10)	0.94 (0.87–1.03)	0.149
Job strain	20.4	20.4	1.00 (0.82–1.21)	19.6	21.7	1.11 (0.99–1.25)	0.90 (0.72–1.13)	0.325
Low social support at work	60.8	52.8	0.87 (0.77–0.98) *	44.1	43.1	0.98 (0.89–1.07)	0.89 (0.77–1.03)	0.099
Low rewards	60.1	51.5	0.86 (0.74–0.99) *	54.2	60.1	1.11 (1.02–1.21) *	0.77 (0.66–0.91) *	0.007 *
ERI ^1^	28.7	26.9	0.94 (0.77–1.14)	31.7	37.2	1.17 (1.06–1.30) *	0.80 (0.64–0.99) *	0.048 *

* Denotes statistical significance, *p* < 0.05; ^1^ QHES = Quebec Healthy Enterprise Standard; CI = Confidence interval; ERI = Effort-reward imbalance; ^2^ Ratio of PRs (effect of group * time interaction) = PR of more exposed organizations/PR of less exposed organizations; ^3^ *p*-value for group * time interaction test.

**Table 4:** Prevalence (%) and prevalence ratios (PR) of high psychological distress according to organizations’ exposure to interventions in the Management Practices area of the QHES ^1^, before (T1) and after (T2) QHES ^1^ implementation.

Psychological Distress	More Exposed Organizations (n = 5)			Less Exposed Organizations (n = 5)			Net Effect of Interventions	
	T1 %	T2 %	PR (95% CI ^1^)	T1 %	T2 %	PR (95% CI ^1^)	Ratio of PRs ^2^ (95% CI ^1^)	*p*-Value ^3^
High psychological distress	32.2	26.6	0.83 (0.73–0.93) *	28.6	27.3	0.96 (0.88–1.03)	0.86 (0.75–0.998) *	0.048 *

* Denotes statistical significance, *p* < 0.05; ^1^ QHES = Quebec Healthy Enterprise Standard; CI = Confidence interval; ^2^ Ratio of PRs (effect of group * time interaction) = PR of more exposed organizations/PR of less exposed organizations; ^3^ *p*-value for group * time interaction test.

## Supplementary Materials

The following are available online at http://www.mdpi.com/1660-4601/15/3/426/s1, Figure S1, Proportion (%) of participants exposed to interventions in the Management Practices area of the QHES as a continuous variable: Prevalence ratios (PR) of adverse psychosocial work factors before (T1) and after (T2) QHES ^1^ implementation for each organization, adjusted for age, sex and education, Figure S2, Proportion (%) of participants exposed to interventions in the Management Practices area of the QHES as a continuous variable: Prevalence ratios (PR) of high psychological distress before (T1) and after (T2) QHES implementation for each organization, adjusted for age, sex and education, Table S1, Prevalence (%) and prevalence ratios (PR) of adverse psychosocial work factors according to organizations’ exposure to interventions in the Management Practices area of the QHES, before (T1) and after (T2) QHES implementation and adjusted for sex, age and education, Table S2, Prevalence (%) and prevalence ratios (PR) of high psychological distress according to organizations’ exposure to interventions in the Management Practices area of the QHES, before (T1) and after (T2) QHES implementation and adjusted for covariates, Table S3, Sensitivity analyses, Prevalence (%) and prevalence ratios (PR) of adverse psychosocial work factors according to organizations’ exposure to interventions in the Management Practices area of the QHES, before (T1) and after (T2) QHES implementation, among the seven organizations with the more complete measure of intervention exposure, Table S4, Sensitivity analyses, Prevalence (%) and prevalence ratios (PR) of high psychological distress according to organizations’ exposure to interventions in the Management Practices area of the QHES, before (T1) and after (T2) QHES implementation, among the seven organizations with the more complete measure of intervention exposure, Table S5, Sensitivity analyses, Prevalence (%) and prevalence ratios (PR) of adverse psychosocial work factors among the three organizations most exposed to interventions in the Management Practices area of the QHES compared to the three organizations least exposed to interventions in this area, before (T1) and after (T2) QHES implementation, Table S6, Sensitivity analyses, Prevalence (%) and prevalence ratios (PR) of high psychological distress among the three organizations most exposed to interventions in the Management Practices area of the QHES compared to the three organizations least exposed to interventions in this area, before (T1) and after (T2) QHES implementation.

## Appendix A

**Table A1:** Items used to assess participants’ perceived exposure to interventions in the Management Practices area of the QHES in versions 1 and 2 of the T2 questionnaire.

Versions 1 and 2 of the T2 Questionnaire	Response Choices	Dichotomization of Responses
**Version 1: 7 organizations (n = 2192; 86% of study participants)**		
Since the implementation of the “Healthy Enterprise” initiative in my workplace, I have noticed changes with regard to (5 items):	No changes implemented	Not exposed = Participant replied “No changes” and “I do not know” for all 5 items
My workload (number of employees, change of duties, time to do tasks, etc.)	Improved my work situation	
My autonomy (participation in decisions that concern me, choice of working methods, etc.)	Did not change my work situation	
The support I receive from my colleagues (sharing of tools and information, meetings, work committees, etc.)	Deteriorated my work situation	Exposed = Participant replied “Improved”, “Did not change” or “Deteriorated” my work situation to at least one item
The support I receive from my immediate superior (team meetings, individual meetings, etc.)	I do not know	
Recognition of my work (efforts and achievements are recognized, promotion prospects, etc.)		
**Version 2: 3 organizations (n = 368; 14% of study participants)**		
As part of the implementation of the “Healthy Enterprise” initiative in my workplace, I noticed changes in the area of activity (1 item):	None at all	Not exposed = Participant replied “None at all”
	A little	
Work organization and management practices (e.g., autonomy, relations with colleagues and superior, consultation, communication, recognition)	Enough	Exposed = Participant replied “Little”, “Enough”, or “Many”
	Many	

The internal consistency (Cronbach’s alpha) of the 5 items in version 1 of the questionnaire is 0.89.
