# Supplementary material for: Evaluation of the Quebec Healthy Enterprise Standard: Effect on Adverse Psychosocial Work Factors and Psychological Distress
Source: Int J Environ Res Public Health. 2018 Feb 28;15(3):426. doi: 10.3390/ijerph15030426 (PMC5876971; doi:10.3390/ijerph15030426)
Supplement: Supplementary file 1 [file ijerph-15-00426-s001.pdf]

Supplementary Materials for the manuscript: *Evaluation of the Quebec Healthy Enterprise Standard: Effect on adverse psychosocial work factors and psychological distress*

Table S1. Prevalence (%) and prevalence ratios (PR) of adverse psychosocial work factors according to organizations' exposure to interventions in the Management Practices area of the QHES[1], before (T1) and after (T2) QHES[1] implementation and adjusted for sex, age and education

	**More exposed organizations (n=5)**			**Less exposed organizations (n=5)**			**Net effect of interventions**	
	**T1 %**	**T2 %**	**PR (95% CI[1])[2]**	**T1 %**	**T2 %**	**PR (95% CI[1])[2]**	**Ratio of PRs[3] (95% CI[1])**	**p-value[4]**
High psychological demands	34.3	38.4	1.12 (0.85-1.47)	35.8	40.2	1.12 (0.97-1.30)	0.998 (0.74-1.35)	0.985
Low decision latitude	61.0	62.5	1.02 (0.88-1.19)	57.1	61.1	1.07 (0.96-1.19)	0.96 (0.81-1.14)	0.476
Job strain	20.0	22.7	1.14 (0.80-1.62)	17.5	20.8	1.19 (0.96-1.46)	0.96 (0.64-1.43)	0.761
Low social support at work	53.8	47.3	0.88 (0.72-1.08)	50.4	49.6	0.99 (0.85-1.14)	0.89 (0.70-1.13)	0.226
Low rewards	57.8	49.2	0.85 (0.70-1.04)	56.7	62.2	1.10 (0.98-1.23)	**0.78 (0.62-0.97)***	**0.038***
ERI[1]	28.2	26.3	0.94 (0.70-1.25)	**32.3**	**37.6**	**1.16 (1.002-1.35)***	0.80 (0.58-1.11)	0.121

[1] QHES= Quebec Healthy Enterprise Standard; CI= Confidence interval; ERI= Effort-reward imbalance

[2] Adjusted for sex, age (<45, 45-54, ≥55) and education (high school degree or less, college degree, university degree)

[3] Ratio of PRs (effect of group*time interaction) = PR of more exposed organizations / PR of less exposed organizations

[4] p-value for group*time interaction test

*Denotes statistical significance, $p < 0.05$

Table S2. Prevalence (%) and prevalence ratios (PR) of high psychological distress according to organizations' exposure to interventions in the Management Practices area of the QHES[1], before (T1) and after (T2) QHES[1] implementation and adjusted for covariates

**High psychological distress**	**More exposed organizations (n=5)**			**Less exposed organizations (n=5)**			**Net effect of interventions**	
**Models adjusted for:**	**T1 %**	**T2 %**	**PR (95% CI[1])**	**T1 %**	**T2 %**	**PR (95% CI[1])**	**Ratio of PRs[5] (95% CI[1])**	**p-value[6]**
Age, sex and education[2]	30.7	25.5	0.83 (0.68-1.01)	30.1	28.6	0.95 (0.84-1.08)	0.87 (0.69-1.10)	0.162
Age, sex, education, physical activity and fruit and vegetable intake[3]	30.9	25.8	0.84 (0.35-2.01)	30.1	28.6	0.95 (0.57-1.59)	0.88 (0.32-2.42)	0.353
Age, sex, education, physical activity and smoking status[4]	30.9	26.4	0.85 (0.39-1.87)	29.5	28.3	0.96 (0.59-1.56)	0.89 (0.36-2.20)	0.347
Age, sex, education, fruit and vegetable intake and smoking status	30.7	26.3	0.86 (0.37-1.97)	29.6	28.4	0.96 (0.58-1.59)	0.89 (0.35-2.32)	0.377

[1] QHES= Quebec Healthy Enterprise Standard; CI= Confidence interval

[2] Sex, age (<45, 45-54, ≥55) and education (high school degree or less, college degree, university degree)

[3] Physical activity (frequency per week <3, ≥3), fruit and vegetable intake (servings/day <5, ≥5)

[4] Smoking status (smoker, non-smoker)

[5] Ratio of PRs (effect of group*time interaction) = PR of more exposed organizations / PR of less exposed organizations

[6] p-value for group*time interaction test

*Denotes statistical significance, $p < 0.05$

Figure S1. Proportion (%) of participants exposed to interventions in the *Management Practices* area of the QHES[1] as a continuous variable: Prevalence ratios (PR) of adverse psychosocial work factors before (T1) and after (T2) QHES[1] implementation for each organization, adjusted[2] for age, sex and education

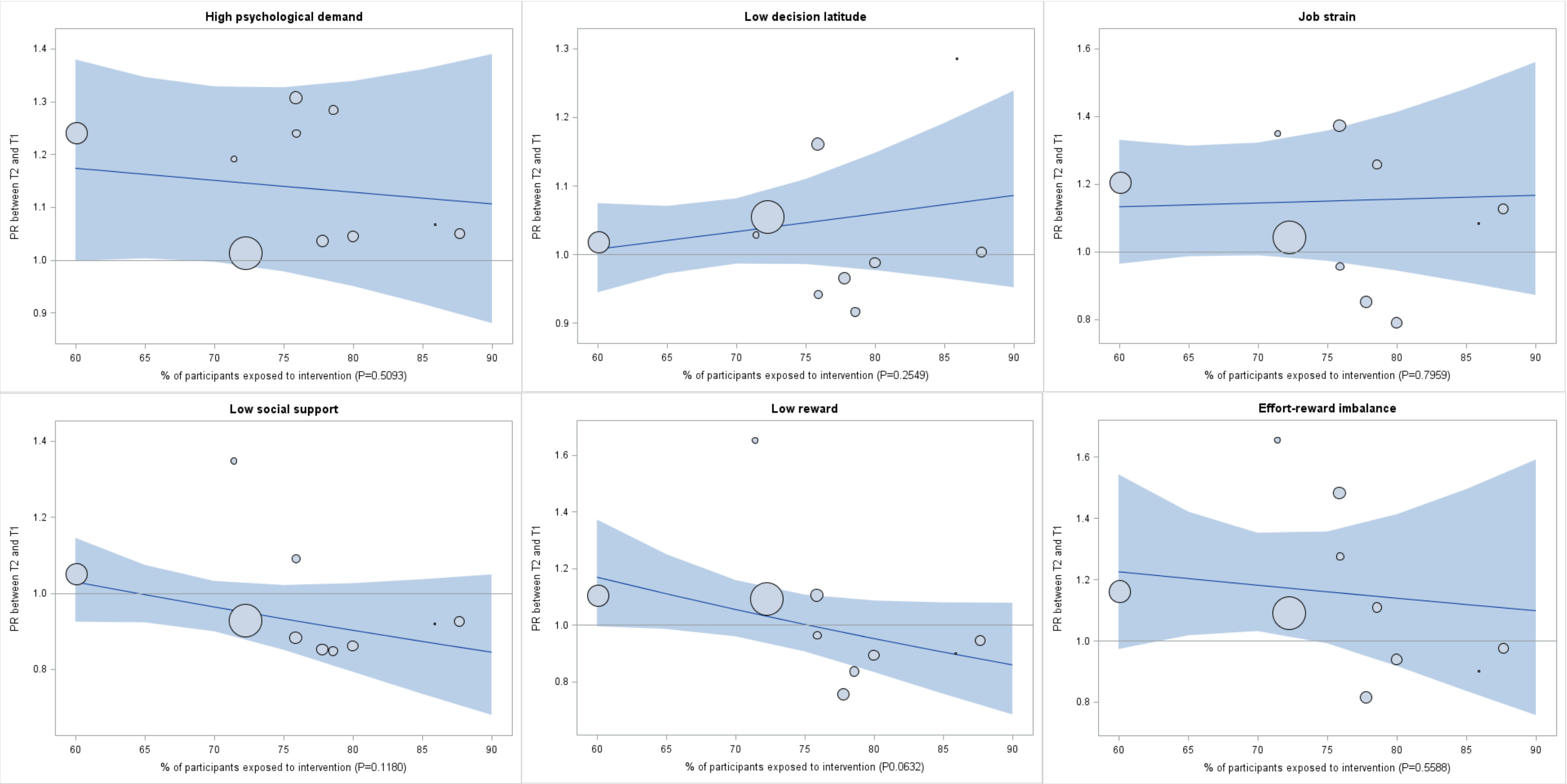

[1] QHES= Quebec Healthy Enterprise Standard; CI= Confidence interval; ERI= Effort-reward imbalance;

[2] Adjusted for age (<45, 45-54, ≥55), sex and education (high school degree or less, college degree, university degree);

Prevalence ratios (PR) of adverse psychosocial work factors before (T1) and after (T2) QHES implementation. PRs were estimated with a repeated measure log-binomial regression where the proportion (%) of participants exposed to interventions in the *Management practices* area of the QHES was considered as a continuous variable. Grey bands represent 95% confidence intervals. The size of the bubbles are proportional to the number of participants in each organization. The horizontal line separates the results between organizations where the prevalence was higher at T2 than at T1 (above the line) and organizations where the prevalence was lower at T2 than at T1 (below the line). *P*-values are for exposition*time interaction test.

Figure S2. Proportion (%) of participants exposed to interventions in the Management Practices area of the QHES[1] as a continuous variable: Prevalence ratios (PR) of high psychological distress before (T1) and after (T2) QHES[1] implementation for each organization, adjusted[2] for age, sex and education

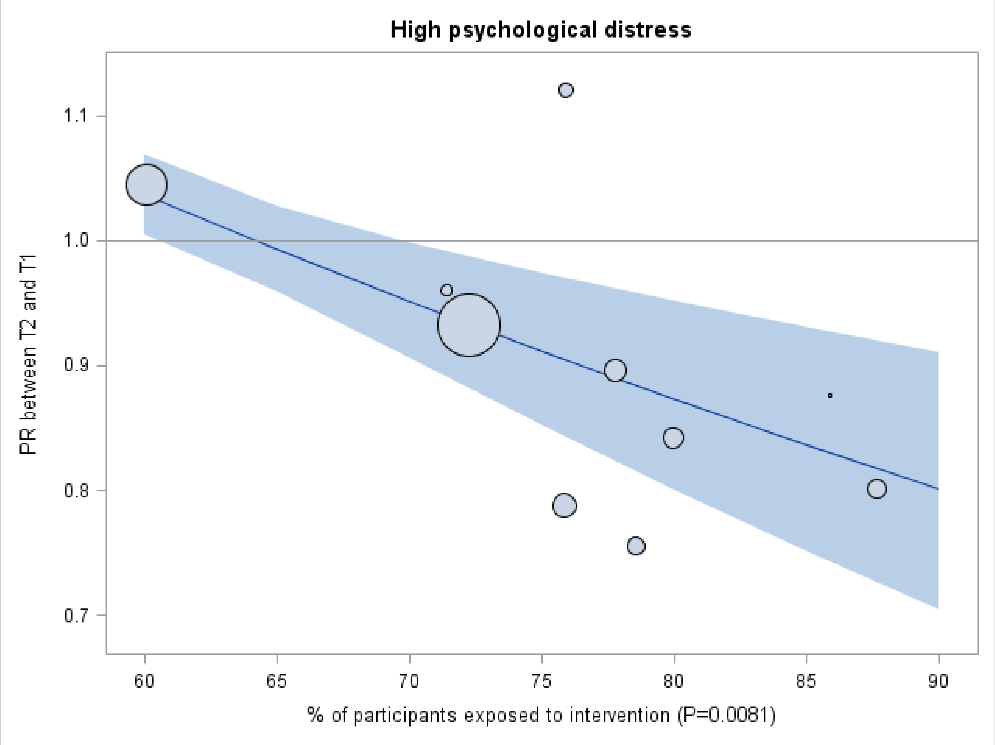

[1] QHES= Quebec Healthy Enterprise Standard; CI= Confidence interval;
[2] Adjusted for age (<45, 45-54, ≥55), sex and education (high school degree or less, college degree, university degree);
Prevalence ratios (PR) of high psychological distress before (T1) and after (T2) QHES implementation. PRs were estimated with a repeated measure log-binomial regression where the proportion (%) of participants exposed to interventions in the *Management practices* area of the QHES was considered as a continuous variable. Grey bands represent 95% confidence intervals. The size of the bubbles are proportional to the number of participants in each organization. The horizontal line separates the results between organizations where the prevalence was higher at T2 than at T1 (above the line) and organizations where the prevalence was lower at T2 than at T1 (below the line). *P*-values are for exposition*time interaction test.

Supplementary Materials for the manuscript: *Evaluation of the Quebec Healthy Enterprise Standard: Effect on adverse psychosocial work factors and psychological distress*

Table S3. Sensitivity analyses; Prevalence (%) and prevalence ratios (PR) of adverse psychosocial work factors according to organizations' exposure to interventions in the Management Practices area of the QHES[1], before (T1) and after (T2) QHES[1] implementation, among the seven organizations with the more complete measure of intervention exposure

	**More exposed organizations (n=4)**			**Less exposed organizations (n=3)**			**Net effect of interventions**	
	**T1 %**	**T2 %**	**PR (95% CI[1])**	**T1 %**	**T2 %**	**PR (95% CI[1])**	**Ratio of PRs[2] (95% CI[1])**	**p-value[3]**
High psychological demands	33.8	37.2	1.10 (0.89-1.36)	38.3	41.8	1.09 (0.96-1.23)	0.97 (0.79-1.29)	0.937
Low decision latitude	70.0	67.9	0.97 (0.91-1.03)	52.7	54.7	1.04 (0.99-1.09)	0.94 (0.86-1.01)	0.078
Job strain	21.2	21.3	1.00 (0.80-1.26)	19.7	21.1	1.07 (0.92-1.24)	0.94 (0.71-1.23)	0.559
Low social support at work	**62.5**	**54.3**	**0.87 (0.77-0.98)***	45.5	44.2	0.97 (0.89-1.06)	0.90 (0.77-1.04)	0.112
Low rewards	**60.7**	**51.8**	**0.85 (0.76-0.96)***	**56.2**	**60.9**	**1.08 (1.01-1.16)***	**0.79 (0.67-0.90)***	**0.006***
ERI[1]	28.7	26.9	0.95 (0.81-1.11)	**31.7**	**37.2**	**1.12 (1.03-1.23)***	0.84 (0.70-1.01)	0.061

[1] QHES= Quebec Healthy Enterprise Standard; CI= Confidence interval; ERI= Effort-reward imbalance

[2] Ratio of PRs (effect of group*time interaction) = PR of more exposed organizations / PR of less exposed organizations

[3] p-value for group*time interaction test

*Denotes statistical significance, $p < 0.05$

Table S4. Sensitivity analysis; Prevalence (%) and prevalence ratios (PR) of high psychological distress according to organizations' exposure to interventions in the Management Practices area of the QHES[1], before (T1) and after (T2) QHES[1] implementation, among the seven organizations with the more complete measure of intervention exposure

	**More exposed organizations (n=4)**			**Less exposed organizations (n=3)**			**Net effect of interventions**	
	**T1 %**	**T2 %**	**PR (95% CI[1])**	**T1 %**	**T2 %**	**PR (95% CI[1])**	**Ratio of PRs[2] (95% CI[1])**	**p-value[3]**
High psychological distress	**32.7**	**27.0**	**0.82 (0.72-0.94)***	28.3	27.7	0.98 (0.88-1.07)	**0.84 (0.72-0.99)***	**0.0397***

[1] QHES= Quebec Healthy Enterprise Standard; CI= Confidence interval

[2] Ratio of PRs (effect of group*time interaction) = PR of more exposed organizations / PR of less exposed organizations

[3] p-value for group*time interaction test

*Denotes statistical significance, $p < 0.05$

Table S5. Sensitivity analyses; Prevalence (%) and prevalence ratios (PR) of adverse psychosocial work factors amongst the three organizations most exposed to interventions in the Management Practices area of the QHES[1] compared to the three organizations least exposed to interventions in this area, before (T1) and after (T2) QHES[1] implementation

	Organizations most exposed to interventions (n=3)			Organizations least exposed to interventions (n=3)			Net effect of interventions	
	**T1 %**	**T2 %**	**PR (95% CI[1])**	**T1 %**	**T2 %**	**PR (95% CI[1])**	**Ratio of PRs[2] (95% CI[1])**	**p-value[3]**
High psychological demands	32.2	33.7	1.04 (0.77-1.42)	39.1	42.4	1.09 (0.95-1.23)	0.96 (0.69-1.34)	0.774
Low decision latitude	75.4	76.8	1.01 (0.96-1.08)	54.6	57.0	1.04 (0.99-1.10)	0.97 (0.90-1.06)	0.461
Job strain	22.2	22.1	1.00 (0.74-1.34)	20.2	22.1	1.10 (0.94-1.28)	0.91 (0.65-1.27)	0.477
Low social support at work	61.4	54.5	0.89 (0.71-1.12)	44.0	43.1	0.98 (0.84-1.14)	0.91 (0.69-1.19)	0.380
Low rewards	60.2	54.9	0.91 (0.70-1.18)	54.6	61.2	1.12 (0.99-1.28)	0.81 (0.61-1.09)	0.119
ERI[1]	27.7	26.1	0.94 (0.68-1.31)	32.7	37.3	1.14 (0.99-1.31)	0.82 (0.58-1.18)	0.215

[1] QHES= Quebec Healthy Enterprise Standard; CI= Confidence interval; ERI= Effort-reward imbalance

[2] Ratio of PRs (effect of group*time interaction) = PR of most exposed organizations / PR of least exposed organizations

[3] p-value for group*time interaction test

*Denotes statistical significance, $p < 0.05$

Table S6. Sensitivity analysis; Prevalence (%) and prevalence ratios (PR) of high psychological distress amongst the three organizations most exposed to interventions in the Management Practices area of the QHES[1] compared to the three organizations least exposed to interventions in this area, before (T1) and after (T2) QHES[1] implementation

	**Organizations most exposed to interventions (n=3)**			**Organizations least exposed to interventions (n=3)**			**Net effect of interventions**	
	**T1 %**	**T2 %**	**PR (95% CI[1])**	**T1 %**	**T2 %**	**PR (95% CI[1])**	**Ratio of PRs[2] (95% CI[1])**	**p-value[3]**
High psychological distress	**31.4**	**25.9**	**0.82 (0.71-0.95)***	28.4	27.4	0.97 (0.90-1.04)	0.85 (0.72-1.004)	0.053

[1] QHES= Quebec Healthy Enterprise Standard; CI= Confidence interval

[2] Ratio of PRs (effect of group*time interaction) = PR of most exposed organizations / PR of least exposed organizations

[3] p-value for group*time interaction test

*Denotes statistical significance, $p < 0.05$
